# Lactoferrin Restores the Deoxynivalenol-Impaired Spermatogenesis and Blood–Testis Barrier Integrity via Improving the Antioxidant Capacity and Modifying the Cell Adhesion and Inflammatory Response

**DOI:** 10.3390/antiox12010152

**Published:** 2023-01-09

**Authors:** Zhaojian Li, Yahui Zhao, Qiufang Zong, Ping Hu, Wenbin Bao, Hao-Yu Liu, Demin Cai

**Affiliations:** Laboratory of Animal Physiology and Molecular Nutrition, College of Animal Science and Technology, Yangzhou University, Yangzhou 225009, China

**Keywords:** deoxynivalenol, lactoferrin, blood–testis barrier, inflammatory response, cell adhesion

## Abstract

Deoxynivalenol (DON) is among the most prevalent contaminants in cereal crops and has been demonstrated to impair male spermatogenesis and induce oxidative stress, testicular apoptosis, and disruption of the blood–testis barrier (BTB). Lactoferrin (LF) is an iron-binding glycoprotein with multifunctions including anti-inflammation and antioxidation. Thus, this study aimed to investigate the effects of LF on the spermatogenesis and integrity of the BTB in DON-exposed mice. Thirty-two male mice were allotted to four groups for a 35-day feeding period: vehicle (basal diet), DON (12 mg/kg), LF (10 mg/d, p.o.), and DON + LF. The results showed that DON induced vacuolization of the spermatogenic epithelium, broke the adhesion junction between Sertoli cells and spermatids established by N-cadherin and induced testicular oxidative stress. LF administration restored sperm production, attenuated the DON-induced oxidative stress and reduced the breakages in adhesion junction. DON exposure enhanced the protein expression of occludin. Transcriptional profiling of the testis observed a disturbance in the expression profiles of cell adhesion and inflammatory response genes, and LF administration reversed these gene expressions. Furthermore, down-regulated signaling pathways, including the apical junction, TNFα signaling via NF-κB, and TGF-β in the DON group were observed. These were restored by LF. Enrichment analysis between DON + LF group and vehicle also confirmed the absence of these pathways. These findings indicated that LF eliminated the DON-induced detriment to spermatogenesis and cell connections between Sertoli cells and spermatids via improving antioxidant capacity and modifying the inflammatory response and cell adhesion genes.

## 1. Introduction

Deoxynivalenol (DON) is a Fusarium-derived trichothecene mycotoxin, one of the most frequently detected mycotoxins in cereals and cereal by-products [1]. Characterized by particularly high resistance to heating and acidic condition, DON can readily contaminate food products and feedstuff formulated with cereals [2,3]. The investigation of more than sixty packages of pelleted dry food for pets showed a 100% detection for DON [4]. Following this, an analysis of twenty-seven distiller’s dried grains with soluble samples, thirty-nine corn gluten feed samples, and thirty mixed feed samples discovered a 100% occurrence of DON, with a range of concentrations varies from 0.12 to 6.2, 0.73 to 12 and 0.15 to 1.2 (mg/kg), respectively [5]. Besides the food, there is growing evidence that DON has become an environmental issue. Wastewater-based epidemiology was performed to monitor eleven wastewater samples from four cities in Italy and Spain, and DON was detected as the being most abundant [6]. Urine samples from swine production workers, and air as well as litter samples from swine farms, all presented mycotoxin contamination, with DON being the most prominent [7]. These extensive exposure pathways make it necessary to investigate the harmful effect of DON, especially reproductive toxicity.

Male reproductive capacity has been reported to be impaired by DON exposure. Gastric incubation of DON in male rats for 28 days elicited adverse effects including decreased epididymal and seminal vesicle weights, sperm reproduction, and serum testosterone concentration, as well as increased serum FSH and LH concentrations [8]. In vitro cultured Leydig cells exposed to DON presented reduced cell viability and capacity of steroidogenesis, as well as elevated ROS production. Additionally, antioxidant agents restored the cell survival and alleviated the generation of ROS, with no beneficial effects on normal progesterone secretion [9]. Further studies, using cell models with the ability of steroidogenesis, elucidated this phenotype. Results showed that expressions of 13 steroidogenic genes were disturbed in the human H295R cell line [10] and protein expressions of steroidogenic acute regulatory (Star) protein and 3β-hydroxysteroid dehydrogenase (3β-HSD) in porcine Leydig cells were blocked by DON exposure [11]. Besides the endocrine-disrupting effects, a recent study also revealed that DON impaired the integrity of the blood–testis barrier (BTB) associated with the reduced expressions of the BTB junction proteins, including occludin, connexin 43, and N-cadherin [12]. The disordered steroidogenesis in Leydig cells and disruption of BTB integrity, the BTB being mainly composed of Sertoli cells, was caused by DON exposure and contributed to the decline in sperm production and the increasing proportion of abnormal sperms.

Lactoferrin (LF) is an iron-binding glycoprotein that is strongly expressed in human and bovine milk. Growing evidence suggests multiple physiological functions of LF, ranging from iron homeostasis and transportation to antimicrobial [13], anti-inflammatory [14], antioxidant and anti-apoptotic effects [15,16]. In asthenoteratospermic men with leukocytospermia, LF administration with other natural antioxidant agents improved semen quality [17]. In vivo and in vitro studies of intestinal barrier functions revealed that LF restored the increased intestinal permeability and impaired barrier function, caused by Aflatoxin M1, via improving the protein levels of tight junction-associated proteins such as claudin-3, occludin, and zonula occludens 1 (ZO-1) [18]. Moreover, LF also presented protective effects on intestinal epithelial barrier dysfunction, induced by infection or inflammation in the cultured epithelial cell. This was paralleled by an inhibition of the enteropathogenic bacterium-induced reduction of claudin-8 expression [19].

Tight junctions also play significant roles in BTB of the testis, and whether LF could restore the impaired BTB functions, antioxidant capacity, and steroidogenesis caused by DON exposure has not yet been studied. In this regard, the present study aimed to assess the protective effects of LF, involving antioxidant and barrier function improvement, in DON-derived testis dysfunctions, a process which will help us to develop novel strategies for coping with the increasing risk of exposure to DON in males.

## 2. Materials and Methods

### 2.1. Experiment Design

Thirty-two male mice (22–28 g) were obtained from the Experiment Animal Centre of Yangzhou University. Mice were raised in a room at constant temperature (25 ± 2 °C) and humidity (50% ± 10%) with a 12-h light–dark cycle and received free access to water and food. DON-contaminated corn was measured and used here as the source of DON. All experimental protocols were approved by the Institutional Animal Care and Use Committee of Yangzhou University (SYXK (SU) 2021-0026).

Mice were conducted with a 5-day adaptation before being allotted by weight to four groups by a stratified random procedure: vehicle group (basal diet, *n* = 8), DON group (basal diet supplemented with 12 mg/kg DON, *n* = 8), LF group (basal diet with oral administration of LF at 10 mg per day, *n* = 8) and DON + LF group (co-treatment of DON and LF with indicated dose above, *n* = 8). All mice were euthanized on day 35 of the feeding period and blood, testis, and epididymis samples were collected. Body weight and the total weight of the bilateral testis were measured. The testis index value was calculated as follows: testis index = testis weight/body weight × 100%. The blood samples were centrifuged at 1000× *g* for 15 min at 4 °C, and the serum was collected and stored at −80 °C. The testes samples were divided into two parts: one side was fixed in 4% paraformaldehyde (Solarbio, P8430, Beijing, China) solution for testicular histological analysis, and the other side was kept at −80 °C for antioxidant status detection, RNA-sequencing (RNA-Seq) analysis, quantificational real-time polymerase chain reaction (qRT-PCR) analyses and Western blot detection.

### 2.2. Antioxidant Parameters in Testicular Tissues

After thawing in normal saline (1:9, *w/v*), testes were mechanically homogenized by a magnetic homogenizer (Hoder, N9548, Beijing, China). Afterward, the homogenate was centrifuged at 3000 rpm for 15 min at 4 °C and the supernatant was collected for protein concentration determination using a commercial BCA (bicinchonininc acid) protein assay kit (Scientific Phygene, PH0326, Fuzhou, China) according to the manufacturer’s protocol.

The concentration of malondialdehyde (MDA) and activities of glutathione peroxidase (GSH-Px), superoxide dismutase (SOD), and total antioxidant capacity (T-AOC) were measured by commercial kits (Jiancheng, A003-1-2, A005-1-2, A001-1-2, A015-1-2, Nanjing, China) following the instructions of the manufacture.

### 2.3. Serum and Testis Hormone Concentration Detection

Concentrations of testosterone in serum and testis homogenate were detected by enzyme-linked immunosorbent assay (ELISA) kits, received from the Institute of Biological Engineering of Nanjing Jiancheng (H090-1-2, Nanjing, China), according to the manufacturer’s protocol. The detection capacity of the kit ranged from 8 nmol/L to 240 nmol/L.

### 2.4. Histology of the Testis and Cauda Epididymis and Immunofluorescence Staining of N-Cadherin

Four testes and cauda epididymis from each group were fixed in 4% paraformaldehyde for 24 h and then immerged through a graded series of ethanol and xylene solutions before being embedded in paraffin blocks. The sections at a 5 μm thickness were obtained by slicing the blocks perpendicular to the longest axis of the testis and cauda epididymis and were subsequently stained with hematoxylin and eosin (HE). After being mounted by coverslips, the sections were detected 200× magnification with a microscope (BX51, Olympus) attached to a video camera (XC 10, Olympus, Tokyo, Japan). Five sections per testis were examined for the cell cluster embedded in the seminiferous epithelium, and at least 20 seminiferous tubules were counted in each section. 

Three testes from each group were fixed in an optimal cutting temperature compound at −20 °C. Then slices at a 7 μm thickness were cut and immerged in 4% paraformaldehyde for 15 min. The sections were then cleaned with PBS and permeabilized with 0.1% Triton X-100 for 10 min. After being blocked with 5% bovine serum albumin (BSA) for 1 h in a moist chamber at RT, N-cadherin was detected with a rabbit anti-N-cadherin antiserum (Proteintech, 22018-1-AP, 1:100, Wuhan, China) in PBS + 5% BSA overnight in the moist chamber at 4 °C. The next day, sections were washed with PBS and incubated with a goat anti-rabbit IgG, conjugated with Alexa Fluor 594 (Abcam, ab150080, 1:300, CB, UK), in PBS + 5% BSA for 1 h at RT. Then, cells were washed and incubated with 3µg/mL 4’,6-diamidino-2-phenylindole (DAPI, a DNA fluorescent dye) for 10 min in a dark area. Finally, sections were washed, and the coverslips dishes were put on the slide with a fluorescence anti-fade reagent (Solarbio, S2100, Beijing, China). Images were obtained using a laser confocal microscope (Thermo Fisher, Waltham, MA, USA) and the Image-Pro Plus software (Carl Zeiss, Jena, Germany).

### 2.5. Sperm Count in the Epididymis and Vas Deferens

The total number of sperm in the cauda epididymis and vas deferens was counted, as described previously [20], with slight modifications. The cauda epididymis and vas deferens were excised together and minced in 0.5 mL PBS. After settling for 10 min, the supernatant was used to perform a sperm count by a standard hemocytometric method.

### 2.6. RNA-Seq Analysis

RNA-seq libraries were obtained using a previously employed method with a small modification [21]. The testis tissue RNA from the vehicle, DON, and DON + LF groups was isolated using the TRIzol reagent (Invitrogen, Carlsbad, CA, USA) and treated with DNase I (RNase-free) (TaKaRa, Dalian, China) to remove genomic DNA. The quality of libraries was validated with an Agilent Bioanalyzer (Agilent Technologies, Palo Alto, CA, USA). Then, sequencing was performed on an Illumina HiSeq 2000 sequencer at BGI Tech (Wuhan, China). The sequence data in a FASTQ format were analyzed using a standard BWA–Bowtie–Cufflinks workflow as reported previously [22]. Sequence reads were mapped to mm9 assembly with BWA and Bowtie software. The Cufflinks package was used for transcript assembly, quantification of normalized gene and isoform expression, and analysis of different expressions. Genes were ranked using Gene Set Enrichment Analysis (GSEA v.4.1) based on the shrunken limma log2 fold changes. The GSEA tool was used in a ‘pre-ranked’ model with default parameters. Gene ontology (GO) analysis was performed using DAVID Bioinformatics Resources. 

### 2.7. Proteins Extraction and Western Blot

Proteins were extracted with a protein extraction kit (Beyotime Institute of Biotechnology, P0027, Nantong, China) according to the manufacturer’s instructions. The proteins extracted from the supernatants were measured with the BCA Protein Assay Protocol and subsequent quantifications of Star (Proteintech, 12225-1-AP, 1:1000, rabbit antibody), 3β-HSD1 (ABclonal, A19266, 1:1000, rabbit antibody, Wuhan, China), Cytochrome P450 Family 11 Subfamily A Member 1 (Cyp11a1, Proteintech, 13363-1-AP, 1:1000, rabbit antibody), N-cadherin (Proteintech, 22018-1-AP, 1:1000, rabbit antibody), occludin (abcam, ab167161, 1:1500, rabbit antibody), connexin43 (ABclonal, A11752, 1:800, rabbit antibody) and β-actin (Beyotime Institute of Biotechnology, AF0003, 1:2000, mouse antibody) were determined by Western blot analysis. Total protein (50μg) was loaded in each lane of 12% PAGE with a MiniProtean Tetra System (BioRad, Hercules, CA, USA) using Precision Plus Protein molecular weight standards (BioRad). The proteins were transferred to nitrocellulose membranes (EMD Millipore Corporation, Billerica, MA, USA). The membranes were incubated with 5% defatted milk powder which was dissolved in phosphate-buffered saline (PBS) for one hour at room temperature to block non-specific binding. Primary antibodies were incubated overnight at 4 °C. Membranes were washed and incubated with HRP-labeled goat anti-rabbit IgG (Huaxingbio, HX2031, 1:5000, Beijing, China), while HRP-labeled goat anti-mouse IgG (Huaxingbio, HX2032, 1:5000) was used for β-actin, followed by visualization using enhanced chemiluminescence (ECL) detection reagents. Bands were scanned in a computer, and their relative intensities were determined by densitometry using Scion Image v. 4.0.2 (Scion Corporation, Frederick, MD, USA). β-actin was used as the cytosolic control. Densitometry was performed with Image J software.

### 2.8. RNA Extraction and qRT-PCR

Total RNA was isolated and validated, as described in RNA-Seq analysis. For each sample, 1 μg of total RNA was reverse-transcribed to cDNA with M-MLV reverse transcriptase (TaKaRa, Dalian, China) and oligonucleotide primers. The housekeeping gene GAPDH and target genes were quantified by real-time PCR on a QuantStudio™ 3 System using a commercial kit (SYBR Premix Ex Taq, TaKaRa, Dalian, China). The gene-specific primers were designed based on the corresponding mRNA sequences with Primer Version 5.0 (Supplementary Table S1). All samples were measured in triplicate. For quantification of real-time PCR results, the threshold cycle Ct was determined for each reaction. Ct values for each gene of interest were normalized to the housekeeping gene. The relative mRNA concentration was calculated using the 2^-ΔΔCt^ method [23] to assess the degree of induction or inhibition, expressed as a “fold difference”, compared to normalized control values. Therefore, all data were statistically analyzed as “fold induction” between treated and vehicle animals.

### 2.9. Statistical Analysis

All the data were subjected to statistical analysis using a completely randomized design. This was performed in accordance with the GLM procedure by GraphPad Prism Version 8.0 software program (GraphPad Software, San Diego, CA, USA). Data were analyzed using a one-way analysis of variance (ANOVA). Statistical difference was accepted when *p* < 0.05. Data were presented as mean ± standard error (SEM).

### 2.10. Data Availability of the Statement

The datasets for RNA-seq in the present study were uploaded to online repositories and could be accessed with the following information: go to https://www.ncbi.nlm.nih.gov/geo/query/acc.cgi?acc=GSE213641 (accessed on 3 January 2023).

## 3. Results

### 3.1. Lactoferrin Administration Increased the Testis Weight and Improved Sperm Production in Deoxynivalenol-Treated Mice

**Figure 1:**
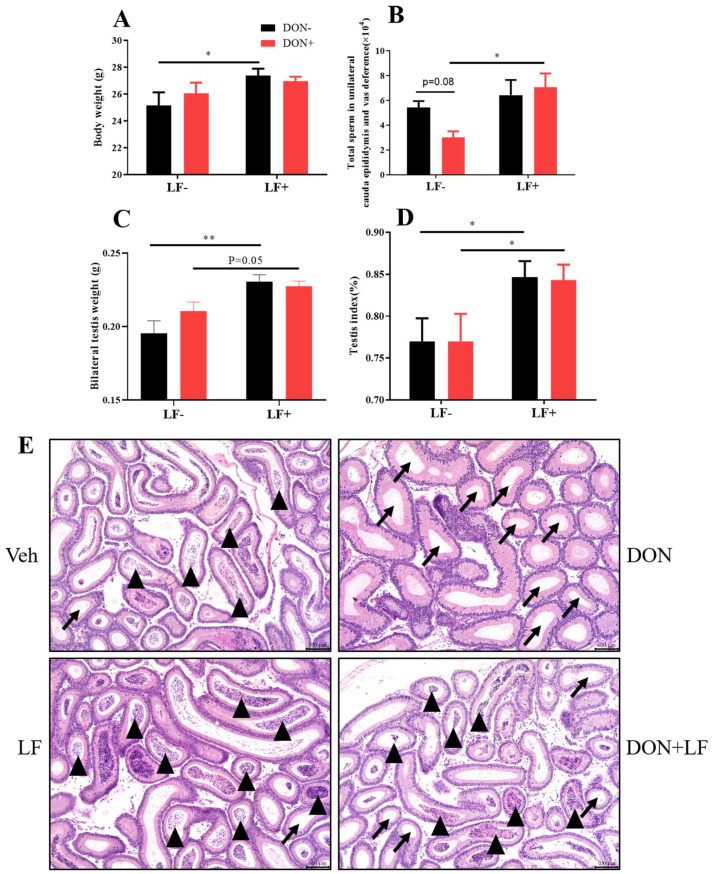
Effects of dietary DON and oral administration of LF on body and testis weight, and sperm count in the epididymis of mice. (**A**) Body weight. (**B**) Total sperm count in unilateral caput epididymis and vas deference. (**C**) Testis weight. (**D**) Index of the bilateral testis. (**E**) Cauda epididymis, histological, arrows indicated tubules with missing sperms and arrowheads indicated tubules with a cluster of sperm. Data were presented as mean ± SEM. For one-way ANOVA, * *p* < 0.05 and ** *p* < 0.01.

Diet supplementation of DON had no effect on body and testis weight, while the oral administration of LF significantly improved the body weight (Figure 1A,C), testis weight and index (Figure 1C,D) compared to the vehicle group. Testis weight and index were also significantly increased in the DON + LF group compared to the DON group. Total sperm count in cauda epididymis and vas deference in DON group exhibited the lowest amount between groups, and LF administration significantly increased the sperm count in DON + LF group compared to the DON group (Figure 1B). Histological analysis of cauda epididymis detected a large number of tubules that were characterized by missing sperms in the DON group, and a substantial number of sperms inside the cauda tubules of the two LF groups also confirmed the counting result of sperm counts (Figure 1E).

### 3.2. Lactoferrin Attenuated the Detrimental Effects of Deoxynivalenol on Spermatogenic Epithelium and Oxidative Status of Testis

**Figure 2:**
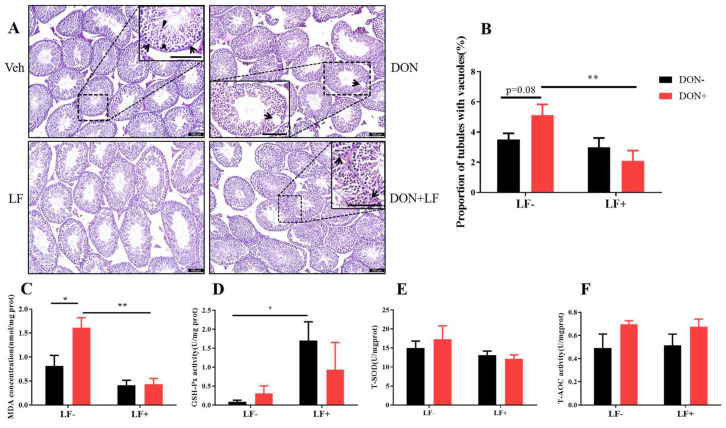
Effects of dietary DON and oral administration of LF on testis histological and oxidative status. (**A**) Testis histological, arrows point to Sertoli cells and the bold arrowhead indicates spermatids and well-developed spermatids with tail extended into the seminiferous tubule matrix (triangle) was observed (enlarged section in Veh); the arrow indicated vacuoles embedded in the epithelium (enlarged section in DON); arrows pointed to Leydig cells in the interstitial tissue of the testis (enlarged section in DON + LF). (**B**) The proportion of tubules with vacuoles. Testicular MDA concentration (**C**), GSH-Px activity (**D**), T-SOD activity (**E**) and T-AOC activity (**F**). Data were presented as mean ± SEM. For one-way ANOVA, * *p* < 0.05 and ** *p* < 0.01.

We next assessed the testicular histopathology by HE staining. In the vehicle and LF groups, testis presented intact seminiferous tubular morphology with a large amount of spermatid clustered in the epithelium: spermatogonia, spermatocytes, spermatids, and mature spermatid were distributed from the basement membrane to the center of the lumen (Figure 2A). While in DON groups, vacuoles embedded among the seminiferous epithelium were revealed and quantitative analysis resulted in a higher proportion of tubules with vacuoles in DON group, which was significantly reduced by LF in DON + LF group (Figure 2B).

Dietary DON significantly increased the testicular MDA content compared to the vehicle group, which was normalized by LF administration in DON + LF group (Figure 2C). LF also significantly improved the testicular GSH-Px activity compared to the vehicle (Figure 2D). No difference in T-SOD and T-AOC activities was revealed between the groups (Figure 2E,F). 

### 3.3. Deoxynivalenol and Lactoferrin Failed to Alter the Testicular Testosterone Production

**Figure 3:**
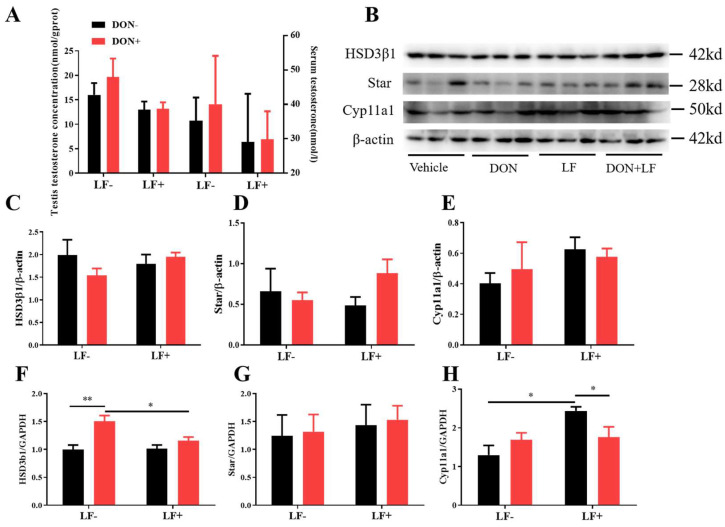
Effects of dietary DON and oral administration of LF on the serum and testicular testosterone concentrations and expressions of steroidogenic genes in the testis. (**A**) Testosterone concentrations in serum and testis; (**B**–**E**) Immunoblotting of HSD3β1, Star, and Cyp11a1 protein expression normalized to β-actin levels and quantification of relative protein density. (**F**–**H**) Relative mRNA expression of steroidogenic genes HSD3β1, Star, and Cyp11a1, normalized to GAPDH expression. Data are expressed as mean ± SEM. For one-way ANOVA, * *p* < 0.05 and ** *p* < 0.01.

Administration of dietary DON and LF resulted in no significant differences in testis and serum testosterone concentrations of mice (Figure 3A). Meanwhile, the protein levels of vital steroidogenesis-related genes, including HSD3β1, Star, and Cyp11a1, showed no significant fluctuation between groups (Figure 3B–E). Intriguingly, up-regulated mRNA transcript levels of HSD3β1 in the testis of DON-treated mice was observed, which was normalized in DON + LF group compared to the DON group (Figure 3F). Meanwhile, oral administration of LF up-regulated gene expression of Cyp11a1 in the testis compared to the vehicle and this elevation was diminished the in DON + LF group (Figure 3H). No difference was revealed in Star gene expression between groups (Figure 3G).

### 3.4. Lactoferrin Improved Integrity of Blood–Testis Barrier Impaired by Deoxynivalenol Exposure 

**Figure 4:**
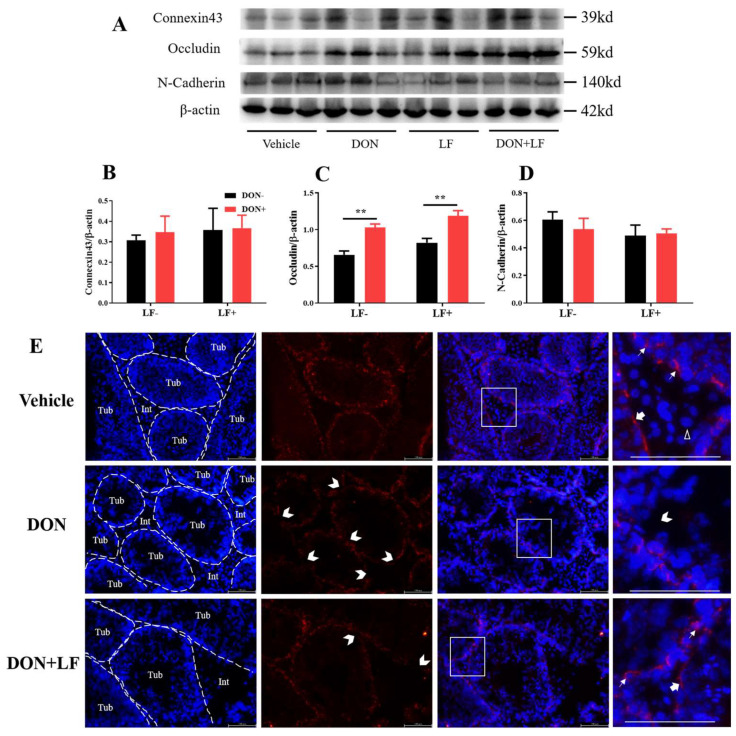
Effects of dietary DON and oral administration of LF on the expressions of BTB junction proteins. (**A**–**D**) Immunoblotting of connexin43, occludin, and N-cadherin protein expression normalized to β-actin levels and quantification of relative protein density. (**E**) Immunofluorescence staining of N-cadherin protein expression. The dashed lines separate the seminiferous tubules (Tub) from the interstitial tissue (Int). The white arrows indicate N-cadherin-positive staining between the spermatids and Sertoli cells. The bold arrows indicate N-cadherin-positive staining at the basement of the spermatogenesis tubules and the white empty arrowhead indicates the N-cadherin-negative Leydig cell. The open arrowheads indicate missing N-cadherin-positive staining sites between Sertoli cells and spermatids in the tubules. Data were presented as mean ± SEM. For one-way ANOVA, ** *p* < 0.01.

To further reveal the mechanism underlying the improvement of LF in terms of DON-induced detrimental effects on histology and sperm production, the expression of the BTB junction proteins was investigated, including connexin43, occludin and N-cadherin (Figure 4A–D). The protein level of occludin was significantly up-regulated in the testis of DON and DON + LF groups (Figure 4C). No difference in protein expressions of connexin43 and N-cadherin was observed. Application of immunofluorescence staining of N-cadherin observed that Sertoli cells and spermatids were significantly stained, represented strong adhesion between these cells (Figure 4E). Leydig cells showed negative staining of N-cadherin. Meanwhile, testis in DON group showed numerous sites embedded in the basement of spermatogenic epithelium that exhibited missingexpression of N-cadherin, indicating breakages in BTB integrity. This phenotype was diminished in the testis of the DON + LF group.

### 3.5. Lactoferrin Reversed Deoxynivalenol-Induced Disturbance of the Genes Involved in Cell Adhesion and Inflammatory Response

**Figure 5:**
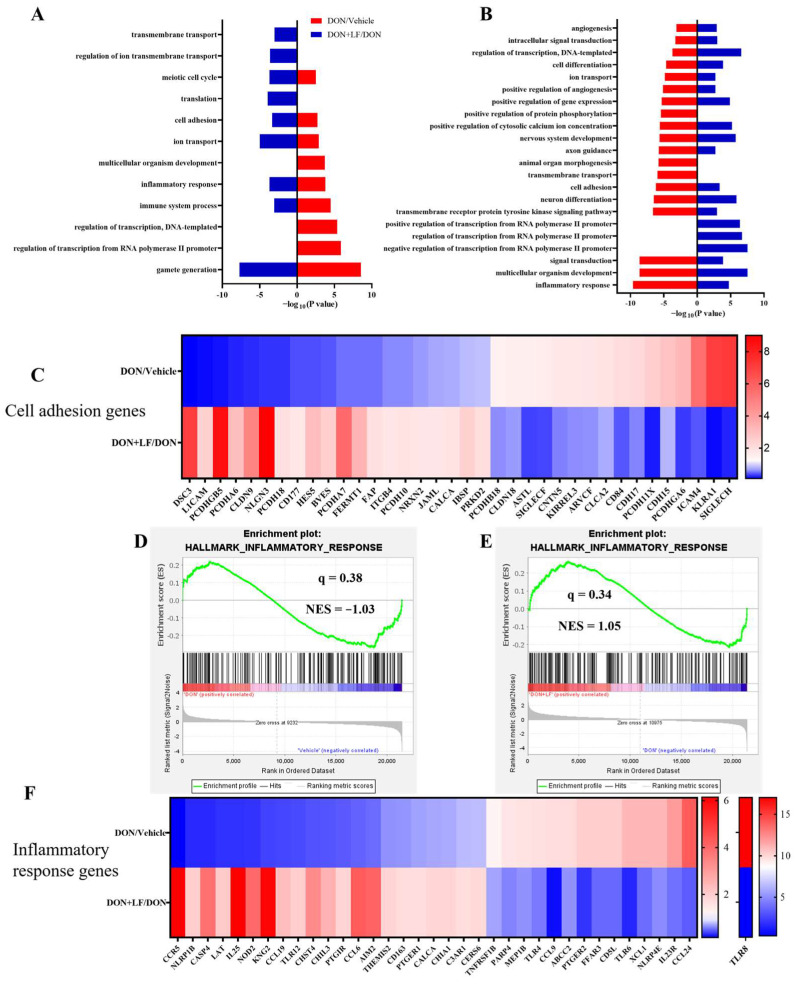
Transcriptional profiling of testes from mice, with diet supplementation of DON and LF. Gene ontology (GO) analysis of the up-regulated genes of the DON group compared to those of the vehicle group coupled with down-regulated genes of the DON + LF group compared to those of the DON group (**A**) and down-regulated genes of the DON group compared to those of the vehicle group coupled with up-regulated genes of DON + LF group compared to those of the DON group (**B**). Hypergeometric testing and Benjamini–Hochberg *p* value correction were applied. Heatmap of mRNA expression (RNA-seq) changes of the cell adhesion genes (**C**) and inflammatory response genes (**F**) in the testes of DON and LF-treated mice. (**D**,**E**) GSEA plots depicting the enrichment of genes involved in the inflammatory response of testes from DON-treated mice compared to the vehicles and DON-LF co-treated mice compared to the DON-treated mice. NES, normalized enrichment score; *q* value indicates false discovery rate *p* value (same for Figure 6).

Transcriptional profiling of the testis was performed to enrich the molecular understanding of the improvement effects of LF on DON-treated testicular dysfunctions. GO analysis of the most down-regulated 3000 transcripts and the most up-regulated 3000 transcripts revealed that diet DON mainly up-regulated gene expressions involving 9 signal pathways, and down-regulated 19 signal pathways in the testis compared to the vehicle. Among these disturbed pathways, 6 pathways that were up-regulated by diet DON presented down-regulation in the testis of the DON + LF group compared to the DON group. Additionally, 16 down-regulated pathways in the testis of the DON group exhibited up-regulation in the DON + LF group compared to that of DON group (Figure 5A,B). Gamete generation was the most enriched up-regulated pathway by diet DON, and it was also the most enriched down-regulated pathway in the testis of the DON + LF group compared to the DON group. Inflammatory response and cell adhesion pathways showed polarization, with some genes down-regulated and others up-regulated in the testis of DON-treated mice, and the polarization was abated by the administration of LF in the testis of the DON + LF group compared to the DON group (Figure 5C,F). The gene-set enrichment analysis (GSEA) also confirmed that the hallmark of the inflammatory response pathway was remarkably altered by diet DON, and LF administration reversed this fluctuation in the inflammatory response pathway in the testis of the DON + LF group compared to that of the DON group (Figure 5D,E). Inflammatory response and cell adhesion pathways were absent in the enriched pathways, a result reached by a comparison between the DON + LF group and the vehicle group by GO analysis and GSEA (Supplementary Figure S2A–E). These reversed pathways, induced by LF administration, play vital roles in protecting the testis from the detrimental effects caused by diet DON.

### 3.6. Lactoferrin Improved the Down-Regulated Signal Pathways Caused by Diet Deoxynivalenol 

**Figure 6:**
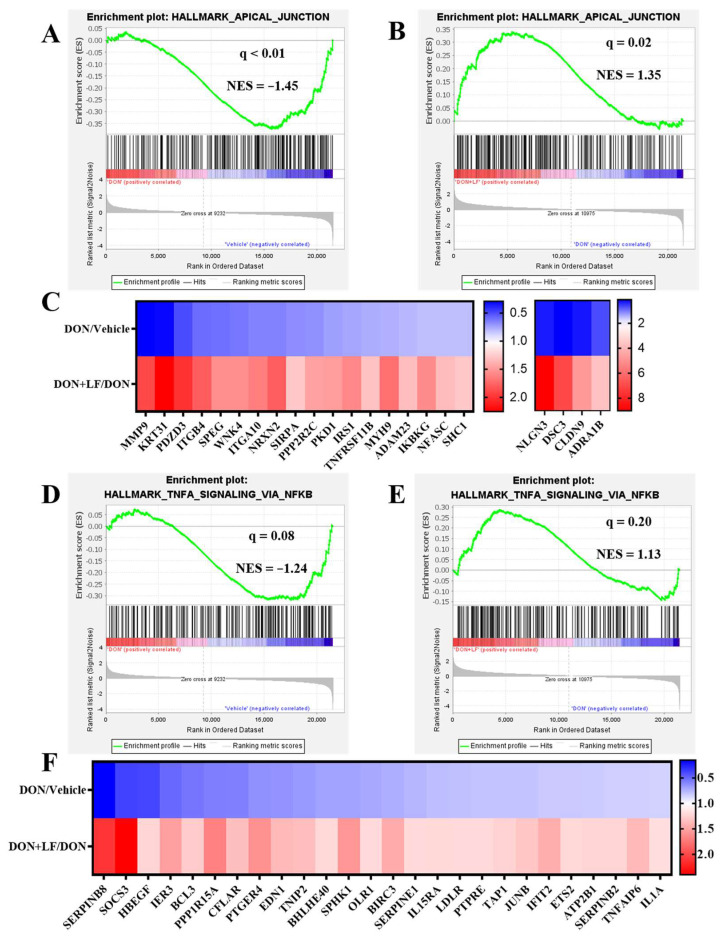
The GSEA plots indicated down-regulated gene expressions involved in apical junction and TNFα signaling via NFκB of testes in the DON group compared to the vehicle and normalizing effects of LF in the DON + LF group compared to the DON group. (**A**,**B**) GSEA plots depicting the enrichment of genes involved in apical junction that were down-regulated in the testes of DON-treated mice compared to the vehicle and up-regulated in the testes of DON-LF co-treated mice compared to those in the DON group. (**C**,**F**) Heatmap of mRNA expression (RNA-seq) changes of the apical junction and TNFα signaling via NFκB genes in the testes of DON and LF treated mice (log2 transformed, normalized to vehicle for DON group, and normalized to DON group for DON + LF group). (**D**,**E**) GSEA plots depicting the enrichment of genes involved in TNFα signaling via NFκB genes that were down-regulated in the testes of DON-treated mice compared to the vehicle and up-regulated in the testes of DON-LF co-treated mice compared to those in the DON group.

In addition to the disturbance pathways observed by GO analysis, GSEA also revealed several pathways that were down-regulated by the addition of diet DON in the testis compared to the vehicle, which dramatically exhibited strong improvement by LF administration in the DON + LF group compared to that in the DON group. These pathways included apical junction (Figure 6A,B), TNFα (tumor necrosis factor-α) signaling via NFκB (Nuclear factor kappa B) (Figure 6D,E), p53 pathway (Supplementary Figure S1A,B), epithelial–mesenchymal transition (Supplementary Figure S1D,E) and TGFβ (transforming growth factor-β) signaling (Supplementary Figure S1G,H). The pathway-focused data analysis showed that large amounts of the apical junction genes (Figure 6C), TNFα signaling via NFκB genes (Figure 6F), p53 pathway genes (Supplementary Figure S1C), epithelial–mesenchymal transition genes (Supplementary Figure S1F) and TGFβ genes (Supplementary Figure S1I) were remarkably down-regulated in DON-treated mice compared to the vehicle, and that all of them were dramatically improved by LF administration in the DON + LF group compared to the DON group. Enriched pathways, by the comparison between the DON + LF group and vehicle, also confirmed the absence of these pathways (Supplementary result Figure S2A–E).

**Figure 7:**
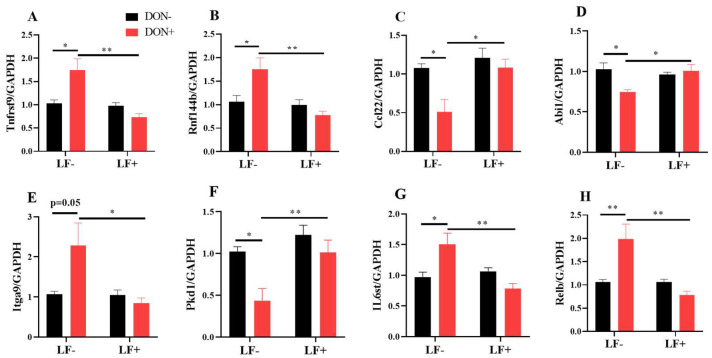
The mRNA transcript level of genes involved in inflammatory response and cell adhesion. (**A**–**H**) Relative mRNA expressions of tumor necrosis factor receptor superfamily, member 9 (Tnfrsf9), ring finger protein 144B (Rnf144b), chemokine (C-C motif) ligand 22 (Ccl22), abl interactor 1 (Abi1), integrin alpha 9 (Itga9), polycystin 1, transient receptor potential channel interacting (Pkd1), interleukin 6 signal transducer (IL6st), and avian reticuloendotheliosis viral (v-rel) oncogene-related B (Relb) normalized to GAPDH expression. Data are expressed as mean ± SEM. For one-way ANOVA, * *p* < 0.05 and ** *p* < 0.01.

The mRNA transcriptional levels of inflammatory response and cell adhesion genes, including Rnf144b, Ccl22, Abi1, Itga9, Pkd1, IL6st and Relb, were further validated by qRT-PCR analysis (Figure 7). Dietary DON significantly increased Tnfrsf9, Rnf144b, Itga9, IL6st and Relb gene expressions, which were normalized by LF administration in DON + LF group (Figure 7A,B,E,G,H). Meanwhile, expressions of Ccl22, Abi1 and Pkd1 were significantly reduced in the DON group compared to the vehicle and these declines were improved to the comparable levels of vehicle by LF in DON + LF group (Figure 7C,D,F).

## 4. Discussion

As the most prevalent contaminants in cereal crops, DON-induced deleterious effects on male reproduction are attracting a growing body of research. Lactoferrin is a glycoprotein, characterized by a strong iron-binding capacity and implicated in a spectrum of physiopathological events related to oxidant and inflammatory processes, which was reported to cure intestinal disorders in various conditions. Our present study revealed the protective effects of LF on dietary DON-induced male reproductive malfunctions. Oral administration of LF restored the spermatogenesis disorder in mice fed a DON inclusion diet, a fact confirmed by an examination of the intact testicular spermatogenic epithelium and the larger amount of epididymis sperms, and eliminated the oxidative stress and breakages in adhesion junction caused by DON treatment. The application of comparative genomics identified that LF reversed the disturbance in the expression of cell adhesion and inflammatory response genes, and improved the down-regulated signal pathways. These include the apical junction, TNFα via NFκB, p53 pathway, and TGFβ signaling in the testes of DON-treated mice. 

As widespread natural contaminants, mycotoxins are the most poisonous biological toxins in food pollution [24]. An increased occurrence of these mycotoxins in the environment contributes to an increase in male infertility [25]. DON with more than 98% purity exposure at doses ranging from 1.2 mg/kg to 4.8 mg/kg body weight in distilled water, reduced mice testis weight and sperm production, as well as increasing sperm malformation rate and damaging the morphological structure of seminiferous epithelium [12,26]. In our present study, moldy corn with a certain DON concentration was used to make the formulation of DON-contaminated diets, of which the dosage referred to a previous study [27]. Even though no decline in body and testis weight was observed in DON-treated mice, numerous azoospermic tubules and reduced sperm amounts in the epididymis were revealed in our result. Mice exhibited improved body weight, testis index, and sperm production with oral administration of LF. Additionally, they showed a lower amount of epididymal azoospermic tubules and a less intact testicular morphology with a lower proportion of seminiferous tubules with vacuoles. 

One possible mechanism underlying DON-induced toxicity is oxidative stress, which is an imbalance between the production of reactive oxygen species/reactive nitrogen species and the antioxidant capacity [28]. Oxidative stress contributed to various organ toxicity caused by in vivo DON exposure including hepatotoxicity, immunotoxicity, and intestinal toxicity [29,30,31]. The testis is also vulnerable to DON, and mice with oral administration of DON showed abundant accumulation of ROS and MDA, accompanied by reduced activities of antioxidant enzymes in the testis [26]. The present study confirmed the DON-induced oxidative stress by a large amount of MDA present in the testis. LF was proven to be a powerful antioxidant [32]. Using a UV irradiation-H2O2 system in human gut epithelial cells, it was observed that LF possessed direct OH- and O2 scavenging potential independent of its iron binding capacity [33]. Here we observed that LF administration markedly declined MDA concentration in the testis of the DON-treated mice with no effect on the activities of antioxidative enzymes, which confirmed LF as a natural antioxidant.

Another major function of the testis is steroidogenesis. This is performed with a series of enzymatic reactions catalyzed by four steroid synthases in Leydig cells including Cyp11a1, HSD3β1, Cyp17a1, and HSD17β1, with cholesterol as the substrate [34]. The main product of steroidogenesis in males is testosterone, and disruption of testosterone biosynthesis results in spermatogenic arrest and male infertility [35]. Previous studies have documented blocked steroidogenesis with reduced serum testosterone concentration after DON administration in rats and mice [8,12]. In vitro studies using granulosa cells indicated that the effects of DON on steroidogenesis were contradictory. These effects resided in the species and dosage that were used [36]. Even though it was observed that DON inhibited the testicular gene transcripts of P450scc, 3β-HSD, P450c17, and 17β-HSD in a dose-dependent manner in mice [12], our present study revealed no difference between serum and testicular testosterone contents as well as testicular protein levels of HSD3β1, Star and Cyp11a1 in DON-treated mice. Meanwhile, LF administration also resulted in no effects on the testosterone production and protein expression levels of the three steroidogenic genes, which might be due to the phenotype that LF, as an antioxidant here, would not improve steroidogenesis in Leydig cells as identified previously [9]. Variation in mRNA levels of HSD3β1 and Cyp11a1, coupled with unchanged protein levels of these two genes, may be attributed to the disrupted translation in the ribosome caused by DON [37].

The blood–testis barrier (BTB), a physical barrier constructed by Sertoli cells, partitions the seminiferous epithelium into two distinct compartments—basal and adluminal. BTB is responsible for sequestering germ cells residing in the adluminal compartment from the circulatory and lymphatic system and, together with local immune suppression, providing an immuno-privileged environment for the completion of spermatogenesis [38]. It is created by the tight junctions (TJs), ectoplasmic specializations (ESs), desmosomes, and gap junctions (GJs) that are present between Sertoli cells [39]. As the most important component of the BTB, TJs restrict the movement of metabolites with selective permeability and the key structural proteins of TJs are occludin, zona occludens (ZO), and claudins [40]. Connexin43 is the most dominant GJs protein within the seminiferous epithelium, which plays a vital role in mammalian spermatogenesis by allowing for direct cytoplasmic communication between neighboring testicular cells and for the maintenance of the homeostasis of the BTB via its effects on tight junction reassembly [41,42]. ESs, a testis-unique anchoring junction, is essential for Sertoli–germ cell communication to support all the phases of germ cell development and maturity, and N-cadherin is the main component of ESs [43]. Given the crucial roles of the BTB in spermatogenesis, a malfunction of sperm production is usually coupled with the disruption of the BTB. However, inconsistent results are described in reports on the BTB protein expressions in animals with impaired spermatogenesis. It was documented that DON and fine particulate matter exposure reduced sperm production and disrupted BTB integrity, with declined protein expressions of occludin, connexin 43, and N-cadherin in mice and rats, respectively [12,44]. In contrast, studies on zearalenone and nano-sized titanium dioxide in mice observed disruption in BTB integrity with increased BTB protein expressions such as connexin 43, claudin-11, and ZO-1 [45,46]. Even though our present study revealed that DON improved the occludin protein expression levels, immunofluorescence staining of N-cadherin revealed breakages in adhesion junction between Sertoli cells and spermatid cells due to DON exposure. This phenomenon may be a compensatory scheme whereby the up-regulation of BTB proteins to maintain the spermatogenesis of testicular tissue that was impaired by DON exposure, as identified by the testis histological and immunofluorescence staining. LF administration reduced the number of breakages among the spermatogenesis epithelium and improved the integrity of adhesion junction between Sertoli cells and spermatid cells that was established by N-cadherin.

Cell adhesion is the ability of a single cell to stick to another cell or an extracellular matrix. Tremendous cell adhesive capabilities exist between germ and Sertoli cells and maintain the integrity of seminiferous epithelium and the general structure of the gonad [47]. With the application of transcriptomic analysis of mRNA expression in the testis, our present study obtained disturbed expression profiles of cell adhesion genes after DON exposure, and LF reversed this disturbance. TJs and adhesion junctions comprise epithelial apical junctions that adhere to neighboring epithelial cells and determine tissue organization [48]. In the testis, apical junctions are involved in constructing BTB. Here we enriched the pathways of the apical junction, itself down-regulated by DON exposure in the testis, indicating a detrimental influence on the BTB function. LF has been reported to support intestinal barrier integrity via restoring the TJ protein expression in a model of short bowel syndrome [49]. An in vitro study on porcine intestinal epithelial cells challenged with lipopolysaccharide (LPS) also observed an improvement in TJ protein expressions by LF [50]. The present result confirmed that LF reversed the down-regulated apical junction pathways caused by DON exposure in the testis, preserving the BTB integral aspects.

The testis is a distinct immune privilege site, where immune cells and testis-specific cells, such as Sertoli cells, Leydig cells, and developing germ cells, all exhibit immunological functions [38]. Here, DON exposure disordered the expression profiles of inflammatory response genes in the testis, which probably was deleterious for spermatogenesis. LF is known to have an anti-inflammatory capacity, and LF administration diminished LPS-induced apoptosis of germ cells in the testis and epididymis [14,51]. The present study verified the anti-inflammatory effects of LF, which reversed expression profiles of inflammatory response genes in the testis of DON-treated mice. Cytokines were reported to regulate barrier function and determine the size of the germ cell population in the seminiferous epithelium [52,53]. As a pro-inflammatory factor, TNF-α protects germ cells from apoptosis in normal human and rat testes [54,55]. TGF-βs contribute to testicular immune privilege through their immunosuppressive activities and the junction restructuring required for spermatogenesis [56,57]. The present study revealed down-regulated gene expression profiles of TNF-α signaling via NFκB and TGF-β signaling in the testis of DON-treated mice, which may contribute to reduced sperm production and impaired BTB integral in DON-treated mice. LF eliminated the decline of the two expression profiles and enhanced the paracrine function of testicular cells to produce enough cytokines to maintain the BTB and promote cell survival during spermatogenesis.

## 5. Conclusions

Taken together, the present results revealed that LF preserved the spermatogenesis and integral aspects of the BTB that were impaired by DON exposure, which was obtained by reduced oxidative stress. Additionally, LF improved the integrity of N-cadherin established adhesion junction between Sertoli cells and spermatid cells, and reversed the signaling pathways, including cell adhesion and apical junction, inflammatory response, and TNF-α signaling via NFκB and TGF-β signaling.

## Data Availability

The data presented in this study are available in the article and supplementary materials.

## Supplementary Materials

The following supporting information can be downloaded at: https://www.mdpi.com/article/10.3390/antiox12010152/s1, Figure S1: The GSEA plots indicated down-regulated gene expressions involved in p53, epithelial–mesenchymal transition, and TGFβ of testis in the DON group compared to the vehicle and normalizing effects of LF in the DON + LF group compared to the DON group; Figure S2: The GO analysis of the up-regulated and down-regulated genes, and GSEA plots depicting the enrichment of genes of the DON + LF group compared to the vehicle group revealed the absent of inflammatory response and cell adhesion genes. Table S1: Primers used for real-time quantitative PCR.
