# Supplementary material for: Lactoferrin Restores the Deoxynivalenol-Impaired Spermatogenesis and Blood–Testis Barrier Integrity via Improving the Antioxidant Capacity and Modifying the Cell Adhesion and Inflammatory Response
Source: Antioxidants (Basel). 2023 Jan 9;12(1):152. doi: 10.3390/antiox12010152 (PMC9855165; doi:10.3390/antiox12010152)
Supplement: Supplementary file 1 [file antioxidants-12-00152-s001.zip › antioxidants-2018105-supplementary.pdf]

Supplementary Table S1 Primers used for real time quantitative PCR

Gene (abbreviation)	Accession numbers	Sequence (5'→3')	Length of product
Star	NM_011485.5	F: ACCTCGGTGCTTTAAGGTGA	166
		R: AGCCACAGTGTTTGCTGAAG	
Cyp11a1	NM_019779.4	F: GGATGCGTCGATACTCTTCTCA	146
		R: GGACGATTCGGTCTTTCTTCCA	
Hsd3β1	NM_008293.4	F: GTACATTTATGGGGAGAGAAGTCC	130
		R: CCAGGCCACATTGCCTACATA	
Cyp17a1	NM_007809.3	F: ATCTCTGGGCACTGCATCAC	156
		R: GAGCCTCTCCAATGCACGAT	
Tnfrsf9	NM_001077509.1	F: TCGCTGCCCTGAGATCGAAA	233
		R: TGTTACAGTTCGGCTGTCCA	
Rnf144b	NM_001170643.1	F: GCTAGAGAGACGCCAAGCAG	275
		R: AGCCTTCTCGGATTGACAGC	
Ccl22	NM_009137.2	F: TTGGCCTCCACCTACTCTCA	116
		R: CGTGCTGCAGACCAAGAAAC	
Abi1	NM_001077190.3	F: CCATGCCTCAGTTGACTCCA	145
		R: GTGGAGTTGGGCTATCAGCA	
Itga9	NM_001113514.1	F: ATCAAGAGTCACCGCTTCGC	90
		R: TGATGCCTTCCTGGTGAGCG	
Pkd1	NM_013630.3	F: AGCTACCTGGACCCATCTGT	122
		R: CAGCACACCAGGCTTTTAGC	
IL6st	NM_010560.3	F: TACCATGGAGCACCTCCCTT	198
		R: CACGAAAGAATACGAGCCCA	
Relb	NM_009046.2	F: TTCGGGCTGTCGGTCCCTA	104
		R: AGGGAAGAGAGGTCACTGGAA	
GAPDH	NM_008084.4	F: TGGAGAAACCTGCCAAGTATGA	135
		R: TGGAAGAATGGGAGTTGCTGT	

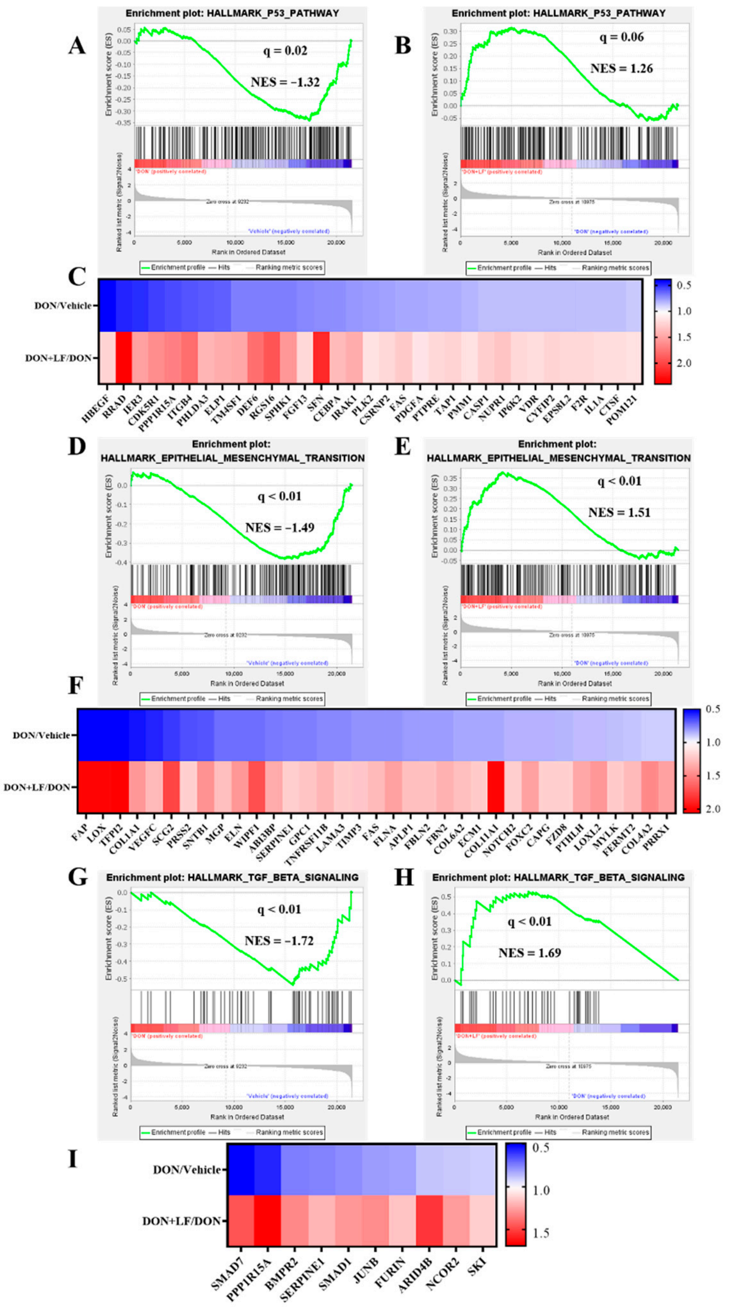

Supplementary Figure S1. The GSEA plots indicated downregulated gene expressions involved in p53, epithelial mesenchymal transition and TGFβ of testes in the DON group compared to the vehicle, and normalizing effects of LF in the DON+LF group compared to the DON group. (A, B) GSEA plots, depicting the enrichment of genes involved in p53 that were downregulated in the testes of DON-treated mice compared to the vehicle, and upregulated in the testes of DON-LF co-treated mice compared to those in the DON group. (D, E) GSEA plots depicting the enrichment of genes involved in epithelial mesenchymal transition that were downregulated in the testes of DON-treated mice compared to the vehicle and upregulated in the testes of DON-LF co-treated mice compared to those in the DON group. (G, H) GSEA plots depicting the enrichment of genes involved in TGFβ that were downregulated in the testes of DON-treated mice compared to the vehicle and upregulated in the testes of DON-LF co-treated mice compared to those in the DON group. (C, F, I) Heatmap of mRNA expression (RNA-seq) changes of p53, epithelial mesenchymal transition and TGFβ in the testes of DON and LF treated mice (log2

transformed, normalized to Vehicle for DON group, and normalized to DON group for DON+LF group). NES—normalized enrichment score; q value—false discovery rate *p* value (same for **supplementary figure S2).**

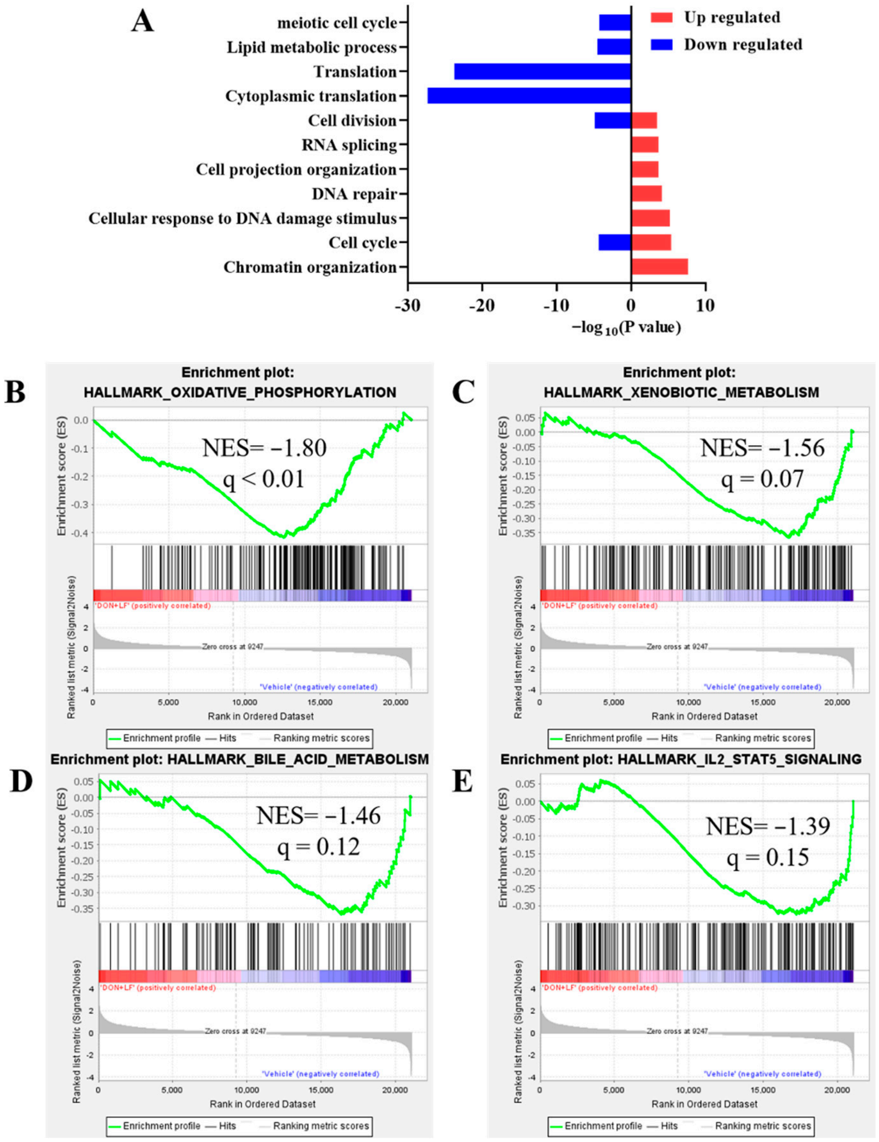

Supplementary Figure S2. Transcriptional profiling of testes from mice in DON+LF group compared to the vehicle group. (A) Gene ontology (GO) analysis of the up-regulated and down-regulated genes of the DON+LF group compared to those of the vehicle group. The hypergeometric test and Benjamini–Hochberg *p* value correction were applied. (B–E) GSEA plots depicting the enrichment of genes involved in the oxidative phosphorylation, xenobiotic metabolism, bile acid metabolism and IL2 STAT5 signaling pathways of testes in mice of the DON+LF group compared to the vehicles.
